# Combating oral biofilms in Nigerian schoolchildren: a synergistic approach using Macrosphyra longistyla extracts and titanium-ferrite nanoparticles

**DOI:** 10.3205/dgkh000551

**Published:** 2025-05-20

**Authors:** Chukwuemeka E. Nwankwo, Adeleke Osho, Adewale Adewuy, Chiagoziem Otuechere, Idowu B. Olawoye, Scott O. Fayemi, Judith U. Oguzie, Jessica Uwanibe, Adedotun F. Adesina, Ernest U. Durugbo, Oluwatobi Adedokun, Damilola Ajisegiri, Ladimeji Akinlawon, Philomena Eromon, Onikepe Folarin, Christian Happi

**Affiliations:** 1 Department of Biological Sciences, Redeemer’s University, Ede, Nigeria; 2 African Centre of Excellence for Genomics of Infectious Diseases (ACEGID), Redeemer’s University, Ede, Nigeria; 3 Department of Chemical Sciences, Redeemer’s University, Ede, Nigeria; 4 Department of Microbiology and Immunology, Schulich School of Medicine and Dentistry, Western University, London, Ontario, Canada; 5 Department of Biological Sciences and Industrial Biotechnology, Caleb University, Imota, Lagos, Nigeria

**Keywords:** biofilm-producing bacteria, Pseudomonas aeruginosa, Aeromonas caviae, Proteus mirabilis, Serratia marcescens, Macrosphyra longistyla nanoparticle complex, minimum inhibitory concentration

## Abstract

**Introduction:**

The burden of infectious and non-infectious debilitating diseases of oral etiology is common in developing countries. The pathogenicity of oral infectious diseases is believed to be exacerbated by the uncontrolled progression of biofilm-producing bacteria. In contemporary research endeavours, there is a proposition to utilize anti-infective compounds in the control of biofilm-induced infections. This research was carried out to isolate and control biofilm-producing bacteria using anti-infective nanoparticles and a plant extract.

**Methods:**

Biofilm-producing bacteria were isolated and characterized using microbiological techniques and next-generation sequencing. Antimicrobial susceptibility testing and minimum inhibitory concentration were determined using titanium ferrite (TF) coupled with *Macrosphyra* *longistyla* plant extracts. Bioactive antimicrobials were analyzed by Fourier-transform Infrared (FTIR) spectroscopy. The surface morphology was determined using a scanning electron microscope (SEM), and toxicological properties were characterized on adult Wistar rats.

**Results:**

Biofilm-producing bacteria isolated and sequenced in this study are *Pseudomonas aeruginosa, Aeromonas caviae, Proteus mirabilis* and *Serratia marcescens*.

The plant extracts coupled with nanoparticles were found to be more bioactive against the biofilm producers than either the plant extracts or the nanoparticles alone. The MICs observed here showed these complexes to be more bioactive against the pathogens in lower concentrations compared to that observed in similar studies.

FTIR revealed that the bands at around 3,000–2,800 cm^–1^ correspond to C–H stretching vibrations. The bands at around 1,700–1,600 cm^–1^ corresponded to C=O stretching vibrations. The bands at around 1,500–1,400 cm^–1^ corresponded to N–H bending vibrations. The presence of these functional groups suggests that *Macrosphyra longistyla* doped with TF nanoparticles (MSLNP) is a complex compound that contains a variety of different chemical groups.

Histology revealed no significant derangements observed in the histoarchitecture of experimental groups. This suggests that the compound shows potential as antimicrobial therapy in battling bacterial oral biofilms. It is recommended that the compound undergo further testing in the drug design process.

## Zusammenfassung

**Einführung:**

Die Belastung durch infektiöse und nicht-infektiöse, zur Invalidität führende Krankheiten oraler Ätiologie ist in Entwicklungsländern weit verbreitet. Man geht davon aus, dass bereits bei Kindern, ausgehend von oralen Infektionen mit unkontrollierter Ausbreitung von Biofilm bildenden Bakterien, Mikroorganismen auf innere Organe des Körpers übergehen und im Erwachsenenalter lebensbedrohliche Krankheiten verursachen können. Die meisten von ihnen sind aggregierende Bakterien, die Biofilme bilden, die sich aus einer Pellikelschicht und Peptidoglykan zusammensetzen und eine Schicht bilden, die sich der Behandlung und/oder Entfernung durch herkömmliche Methoden entzieht und schließlich zur Resistenz gegen antimikrobielle Mittel führt. Ziel dieser Studie war es, orale Bakterien zu identifizieren, die Biofilme bei Schülern aus dem Südwesten Nigerias bilden.

**Methode:**

Die mikrobiologische Diagnostik wurde mittels Next-Generation-Sequenzierung durchgeführt. Der Titan-Ferrit-Komplex mit dem Pflanzenextrakt von *Macrosphyra* *longistyla* wurde mittels Fourier-transformations-Infrared (FTIR) Spektroskopie, die Oberflächenmorphologie elektronenmikroskopisch und die toxikologischen Eigenschaften wurden an Wistarratten untersucht.

**Ergebnisse:**

Als biofilmbildende Bakterien wurden *Pseudomonas aeruginosa, Aeromonas caviae, Proteus mirabilis* und *Serratia marcescens* identifiziert.

Mit Nanopartikeln gekoppelte Pflanzenextrakte waren bioaktiver gegen die Biofilmbildner als die Pflanzenextrakte oder die Nanopartikel allein. Die minimalen Hemmkonzentrationen ergaben, dass diese Komplexe in niedrigeren Konzentrationen bioaktiver gegen die Erreger sind als die in ähnlichen Studien beobachtete Wirksamkeit.

Im Ergebnis der Fourier-Transformations-Infrarot (FTRI)-Spektroskopie entsprechen die Banden bei etwa 3,000–2,800 cm^–1^ den C–H-Streckschwingungen, die Banden bei ca. 1,700–1,600 cm^–1^ den C=O-Streckschwingungen und die Banden bei ca. 1,500–1,400 cm^–1^ den N–H-Biegeschwingungen. Das Vorhandensein der funktionellen Gruppen lässt darauf schließen, dass *Macrosphyra longistyla* gedoped mit Nanopartikeln (MSLNP) ein komplexes Molekül ist, das eine Vielzahl verschiedener chemischer Gruppen enthält. Die histologische Untersuchung ergab keine signifikanten Veränderungen in der Histoarchitektur der Gruppen. Das deutet darauf hin, dass die Verbindung ein Potenzial für eine antimikrobielle Therapie bei der Bekämpfung oraler Biofilme bakterieller Ätiologie aufweist. Es wird empfohlen, den Wirkstoff für die Arzneimittelentwicklung weiter zu bearbeiten.

## Introduction

Microorganisms survive hostile environments by adopting a variety of mechanisms and states which may be initiated by the production of biofilms [1]. The production of biofilms by bacteria found in human hosts poses enormous health risks. Oral biofilm-producing bacteria in children may directly or indirectly impact morbidity, mortality and overall quality of life to varying degrees of disease that may proceed to adulthood [2], [3], [4], [5], [6]. Diseases such as caries and periodontal diseases are caused by biofilm-producing microorganisms which have been found and isolated from children and adults [7], [8], [9], [10], [11]. Biofilm-producing bacteria make up part of the 700 species of bacteria that can be found in the human oral cavity. These microorganisms are responsible for a high number of systemic diseases with public health risk [12], [13]. They may be disseminated to internal organs and can serve as markers of severe diseases, e.g., cancers and heart disease, responsible for up to 10% of global deaths [14], [15]. The problem is further exacerbated by the presence of antimicrobial-resistant forms of these pathogens. Bacteria implicated in oral biofilms include *Streptococcus*, *Proteus (P.)* and *Pseudomonas (P.)* spp. [16], [17]. 

Conventional methods of controlling oral biofilm-producing bacteria vary across regions of the world. In Nigeria, these oral care measures include the use of toothpaste, chew stick and mouthwash. The need for the prevention and control of diseases caused by oral biofilm-producing bacteria relates to the condition observed when caries and periodontal disease reach an advanced stage, requiring the dentist to mechanically remove biofilms from the mouth as a treatment measure [18]. In-vitro antimicrobial treatment measures include the use of plant extracts, nanoparticles, and essential oils. Other measures include non-antibiotic strategies like antimicrobial photodynamic therapy (APDT), cold atmospheric plasma (CAP), and the in-silico data-driven BiofOmics platform [19]. Microorganisms not eliminated by the above-listed methods eventually become part of the host’s microbial identity [20], [21]. 

In the laboratory, oral biofilms are studied using microbiological techniques in conjunction with others based on chemistry, molecular biology and biophysics. Antibiofilm approaches employed in the laboratory include the use of synthetic antimicrobial peptides and antibiotics, plant extracts and other custom-made chemicals such as nanoparticles. Mechanisms that contribute to the growth and spread of bacterial biofilms include contact killing, inhibition of quorum sensing, alterations to the membranes of host cells and peptidoglycan cleavage, inhibition of cell division and dispersion, as well as other methods of evading host defence systems [17].

Nanoparticles show great promise in their use as antibiofilm agents due their small size and properties that can be controlled in the creation of custom-made antimicrobials. Their size, shape and optical properties are controlled during synthesis, and they can be combined with plant extracts as capping agents, enhancing their antimicrobial properties. Reports suggest that nanoparticles can be bioactive not only when they exist singly but also when they are coupled with other molecules, e.g., plant extracts [22], [23], [24]. The use of nanoparticles and plant extracts against oral biofilm-producing bacteria has been demonstrated by Ramzan et al. [25], Moghadam et al. [26] and Ahmed et al. [27]. 

There is a need for more bioactive molecules to slow the progression of antimicrobial resistance; this paper aims to proffer solutions to this end. In this research, we have attemped to ascertain which biofilm-producing bacteria are present in the oral cavity of schoolchildren in southwestern Nigeria using metagenomics. Functional profiles were determined molecularly by identifying the molecular markers of drug resistance in the bacteria. Their antimicrobial susceptibility to *Macrosphyra longistyla* extracts, titanium ferrite nanoparticles, and the extracts coupled with the TF nanoparticles were examined. The toxicity patterns of the active complex were determined using Wistar rats. This was followed by advanced characterisation of the complexes.

## Materials and methods

### Ethical approval

Ethical approval was obtained from Redeemer’s University Directorate of Research, Innovation and Partnerships, Osun Local Government, Lagos Education Board, Ibadan State Universal Basic Education Board, Nigeria. Samples of schoolchildren were obtained after obtaining informed consent from parents or guardians.

Schoolchildren aged 4–14 years from 5 schools (n=50 each) in Lagos, Osun and Oyo states of Nigeria were included. Every participant was given 10 ml of sterile potable drinking water to gargle. After gargling for 10 seconds, they were asked to expectorate the mouth-rinsing liquid into sterile sample bottles which were corked, labelled and transported to the laboratory ensuring sample integrity and asepsis. 

### Bacteria

#### Culture and isolation

Bacteria were cultured by the pour plate method. This involved the addition of 1 ml of the gargled water sample onto a sterile petri dish, to which 15 ml of sterile molten nutrient agar was added, swirled clockwise and anti-clockwise and allowed to set. Labelled petri dishes with samples were then incubated at 37oC for 18–24 hours. After incubation, the colony forming units (CFU) were enumerated and distinct representative colonies were inoculated onto brain-heart infusion agar with Congo red as the indicator. Black colouration after 24-hour incubation indicated biofilm formation. Biofilm-forming isolates were then subcultured and stored on agar slants in bijou bottles for further analyses. 

#### Morphological and biochemical characterisation of isolates

Distinct colonies were observed for their morphological and biochemical characteristics [28]. Tests include Gram staining, lactose fermentation, mannitol fermentation, glucose fermentation, maltose fermentation, sucrose fermentation (change in colouration after 24–48 hours indicates positive result, and gas bubble indicates that the microorganism is aerobic), oxidase, catalase, citrate utilization, indole, motility, hydrogen sulphide, test for growth on Kligler’s iron agar (KIA), urease, methyl red and Vogues Proskauer tests.

#### DNA extraction, quantification and library preparation

DNA was extracted from the biofilm-producing bacterial isolates using the Zymo Research Quick-DNA^TM^ Miniprep Plus Kit following the manufacturer’s instructions. DNA quantification was subsequently carried out using a Qubit dsDNA HS kit and measured with a Qubit 3.0 fluorimeter (ThermoFisher). DNA size distribution was determined using an Agilent 2200 Tapestation system with genomic screentapes (Agilent Technologies). DNA purity was determined using a NanoDrop One Spectrophotometer (ThermoFisher). Nanogram (flex) was used after which fragmentation (amplification and indexing) and library preparation was carried out followed by tagmentation to add indices. Library preparation was carried out using Nextera DNA flex library preparation kit. Water was used as the negative control, and identifiers (IDs) were recorded on sample sheets, after which concentration with sample IDs were obtained and labelled.

#### Whole-genome sequencing and molecular identification of biofilm-producing bacteria

DNA was extracted from the biofilm-producing bacterial isolates using the Zymo Research Quick-DNA^TM^ Miniprep Plus Kit following the manufacturer’s instructions. DNA purity was determined using a NanoDrop One Spectrophotometer (ThermoFisher). DNA quantification was subsequently carried out using a Qubit dsDNA HS kit and measured with a Qubit Flex fluorimeter (ThermoFisher). DNA size distribution was determined using an Agilent 2100 BioAnalyzer with a High Sensitivity DNA chip (Agilent Technologies). Fragmentation (amplification and indexing) and library preparation was carried out followed by tagmentation to add indices. Library preparation was carried out using the NexteraXT library preparation kit (Matranga et al., 2016). Water was used as negative control, and identifiers (IDs) were recorded on sample sheets, after which concentration with sample IDs were obtained and labelled. Whole genome sequencing (WGS) was conducted on an Illumina NextSeq 2000 system using Illumina’s NextSeq 2000 P3 Reagents (300 cycles). 

Raw FASTQ reads were processed with the TheiaProk Illumina pipeline (https://theiagen.notion.site/TheiaProk-Workflow-Series-cc66a9dc42a144a789990935465bc9ff) for quality assessment, *de novo* genome assembly, genome annotation, taxonomic characterisation, and antimicrobial resistance prediction of the bacterial genomes. Isolates that did not pass the quality assessment criteria were excluded from further analyses, as they were deemed contaminated or mixed isolates.

### TiFe_2_O_4_ particles

#### Preparation

TiFe_2_O_4_ particles were prepared as previously reported [29]. Briefly, 200 mL solutions of Ti(NO_3_)_4_ (0.2 M) and FeCl_3_.6H_2_O (0.4 M) were stirred in a conical flask for 1 h at 70°C. During the stirring, oleic acid (10 mL) was added as a capping agent to control the particle growth. Ammonia solution (10 mL) was added after 1 h of stirring to bring the reaction solution to a pH range of 9–11 to precipitate the TiFe_2_O_4_ particles from solution. This was further stirred for 30 min before the reaction was terminated. The TiFe_2_O_4_ particles in reaction solution was filtered (using Whatman paper) and washed several times with deionized water. The filtrate was air dried overnight and later transferred to a muffle furnace for 12 h at 600°C.

#### Extraction process

Ethanol and aqueous extracts of *Macrosphyra longistyla* leaves and stem bark were obtained by air drying the plant parts in shade at ambient temperature and blending to obtain fine ground powder [30]. These powders were then separately extracted using ethanol or distilled water in a cold extraction process by soaking them in a 2-L conical flask in the dark for 24 hours. The extracts obtained were concentrated using a rotary evaporator. Extracts obtained were then labelled and stored in a refrigerator for further use.

#### Preparation of TiFe_2_O_4_ doped Macrosphyra longistyla extracts

TiFe_2_O_4_@MLSBAE and TiFe_2_O_4_@MLSBEE were prepared by dispersing TiFe_2_O_4_ (1 g) in *Macrosphyra longistyla* aqueous stem-bark extract (100 mL) and *Macrosphyra longistyla* ethanol stem-bark extract (100 mL), respectively. Similarly, TiFe_2_O_4_@MLLAE and TiFe_2_O_4_@MLLEE (MSLNP) were prepared by dispersing TiFe_2_O_4_ (1 g) in *Macrosphyra longistyla* aqueous leaf extract (100 mL) and *Macrosphyra longistyla* ethanol leaf extract (100 mL), respectively. The TiFe_2_O_4_ dispersed solution was kept at 60°C while stirring for 1 h. The resulting product was filtered and washed several times with extraction solvent (ethanol for TiFe_2_O_4_@MLSBEE or TiFe_2_O_4_@MLLEE and distilled water for TiFe_2_O_4_@MLSBAE or TiFe_2_O_4_@MLLAE). The resulting TiFe_2_O_4_@MLSBAE, TiFe_2_O_4_@MLSBEE, TiFe_2_O_4_@MLLAE and TiFe_2_O_4_@MLLEE were air dried overnight.

#### Characterisation of TiFe_2_O_4_, TiFe_2_O_4_@MLSBAE, TiFe_2_O_4_@MLSBEE, TiFe_2_O_4_@MLLAE and TiFe_2_O_4_@MLLEE

The functional groups present in TiFe_2_O_4_, TiFe_2_O_4_@MLSBAE, TiFe_2_O_4_@MLSBEE, TiFe_2_O_4_@MLLAE and TiFe_2_O_4_@MLLEE (MSLNP) were determined by FTIR (Shimadzu FTIR-8400S). The surface morphology was determined using a scanning electron microscope (SEM) (JOEL Co Japan) to understand the surface structure, while the x-ray diffraction pattern of the particles was analysed using an x-ray diffractometer (in the range 5 to 90°) at 2θ. 

#### Antimicrobial efficacy

The biofilm-producing bacteria were cultured on Mueller-Hinton agar containing plant extracts and the nanoparticles complex. Each isolated bacterial colony was aseptically mixed with normal saline to make a suspension comparable to the 0.5 McFarland standard, which represents approximately 1.5x10^8^ CFU/ml of bacteria. Gram-positive and Gram-negative bacteria were inoculated and incubated at 37°C for 18–24 hours. The agar well diffusion method was used, inoculating 6-mm wells with 100 microlitres of suspensions of biofilm-forming bacteria. Zones of inhibition recorded and susceptible isolates with zones of inhibition >15 mm were selected for minimum inhibitory concentration (MIC) determination and further characterisation. 

Antimicrobial susceptibility screening was conducted on the *Macrosphyra longistyla* aqueous extract (ML@Aqua), *Macrosphyra longistyla* ethanol extract (ML@Etha), TiFe_2_O_4_, TiFe_2_O_4_@MLSBAE, TiFe_2_O_4_@MLSBEE, TiFe_2_O_4_@MLLAE and TiFe_2_O_4_@MLLEE (MSLNP). An initial concentration of 2.50 mg/L of the test samples were used for the susceptibility study via the agar-well diffusion method of the 0.5 MacFarland standard of suspensions of corresponding bacteria as previously reported by Jensen et al. [31]. The MacFarland standard represents the optical density of 1.5x10_8_ CFU/mL of bacteria cells. Zones of inhibition were recorded. Subsequently, MIC was detected using 96-well microplates, and optical densities were recorded at 6-h intervals during incubation (35°C for 24–48 hours) of the bacterial suspensions in each well treated with the antimicrobial, both using an ELISA reader and by visual inspection [32]. The MIC is given as the lowest concentration of the active test sample which can inhibit the growth of the biofilm-producing microorganisms.

### Animal experiments

#### Husbandry

Adult Wistar rats weighing 80–120 g were obtained from the animal facility, Redeemer’s University, Ede Osun State. Rats were fed on a commercial-pellet diet (Ladokun Feeds Ibadan, Nigeria) and drinking water ad libitum, while being maintained under standard laboratory conditions and subjected to a natural photoperiod of 12 h light/12 h dark cycle.

#### Collection of samples

Rats were sacrificed by cervical dislocation and blood samples were collected by cardiac puncture into centrifuge tubes. These were centrifuged at 3,000 g for 10 min in a laboratory centrifuge to obtain the plasma. 

#### Experimental design

Only TiFe_2_O_4_@MLLEE (MSLNP) was used for the animal experiment, because it exhibited the best antimicrobial activity among the test samples. Wistar rats (21) distributed into three groups of seven animals each were treated orally, once daily, for 14 days:

- Group A: Control, received normal saline
- Group B: 5 mg/kg TiFe_2_O_4_@MLLEE
- Group C: 10 mg/kg TiFe_2_O_4_@MLLEE

At the end of treatment, samples were collected for biochemical analysis.

#### Biochemical assays

Plasma concentrations of aspartate aminotransferase (AST), alanine aminotransferase (ALT), alkaline phosphatase (ALP), gamma-glutamyl transferase (GGT), albumin, total cholesterol, urea, creatinine, bilirubin, uric acid, triglycerides (TG), high density lipoprotein (HDL), sodium and potassium were determined using commercially available diagnostic kits (Randox Lab. Limited). 

#### Histology

Livers and kidneys from rats of all the groups were fixed in 10% formaldehyde, dehydrated in graded alcohol and embedded in paraffin. Fine sections were obtained, mounted on glass slides and counter-stained with hematoxylin-eosin (H&E) and Periodic Acid Schiff (PAS) for light-microscopic analyses. The slides were coded and examined by a histopathologist.

## Results

### Characterisation of the isolates

The following species were isolated: *Pseudomonas aeruginosa, Stenotrophomonas maltophilia, Proteus mirabilis, Pseudomonas stutzeri, Serratia marcescens* and *Aeromonas caviae*. The morphological and biochemical characterisation of isolates are given in the supplement (Table 1 (Tab. 1) and Table 2 (Tab. 2)). Table 1 (Tab. 1) shows the morphology of the bacterial isolates obtained in this study. Table 2 (Tab. 2) presents the biochemical characterisations of the isolates subjected to biofilm production assays after they were isolated from culture media containing phenotypically diverse oral bacteria from the test participants.

### Genomic identification, molecular characterisation and antimicrobial gene expression

The results indicate the successful identification and characterisation of bacterial species in the experimental organisms. Notably, the samples of *P. aeruginosa* exhibited consistent high-quality sequencing, as evidenced by high BUSCO scores (Table 3 (Tab. 3)).

Understanding the antimicrobial resistance (AMR) profiles of bacterial isolates is essential for devising effective strategies to combat infectious diseases. This study investigates the presence of AMR genes and subclasses in experimental organisms, focusing on their resistance to various antimicrobial agents. The presence of diverse AMR genes and subclasses in the experimental organisms highlights the complexity of antimicrobial resistance. The observed resistance profiles provide valuable insights for further studies on the evolution and dissemination of resistance mechanisms (Table 4 (Tab. 4)).

### Antimicrobial susceptibility testing and minimum inhibitory concentration (MIC)

Results of the antimicrobial susceptibility testing of the complexes used in this study are given in Table 5 (Tab. 5) and Table 6 (Tab. 6). The MIC is given as the lowest concentration of compound that inhibits visible bacterial growth. MICs were only determined for bioactive compounds eliciting zones of inhibition ≥15 mm. 

### Toxicological data

The effects of MSLNP on organ weights and relative organ weights are shown in Table 7 (Tab. 7). There were no significant changes (p<0.05) in the liver and kidney weights of the rats. Similarly, MSLNP did not produce any significant changes in the relative liver and relative kidney weights when compared to the control. The relative weight is given as the ratio of organ to body weight of the harvested organs upon sacrifice after administration of the test compound.

**Figure 1:**
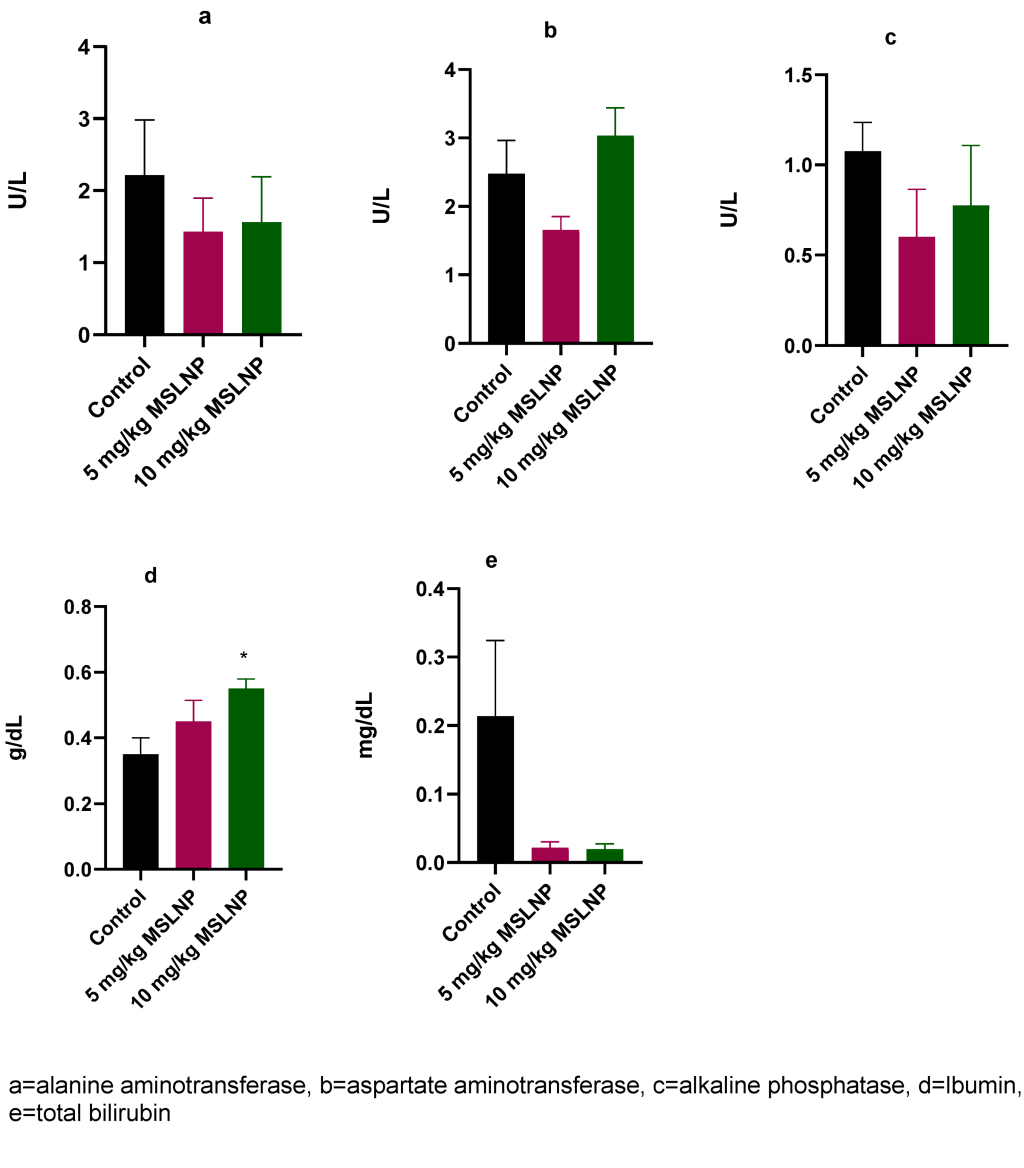
Effects of MSLNP on plasma liver function parameters (mean and standard deviation of 7 animals, *values differ significantly from the control P<0.05)

Figure 1 (Fig. 1) shows that administration of MSLNP did not elicit any significant changes in the plasma levels of ALT, AST, ALP and total bilirubin in antimicrobially treated rats compared to control rats. However, there was significant increase (p<0.05) in plasma albumin concentration in rats that received 10 mg/kg MSLNP when compared to the control group.

**Figure 2:**
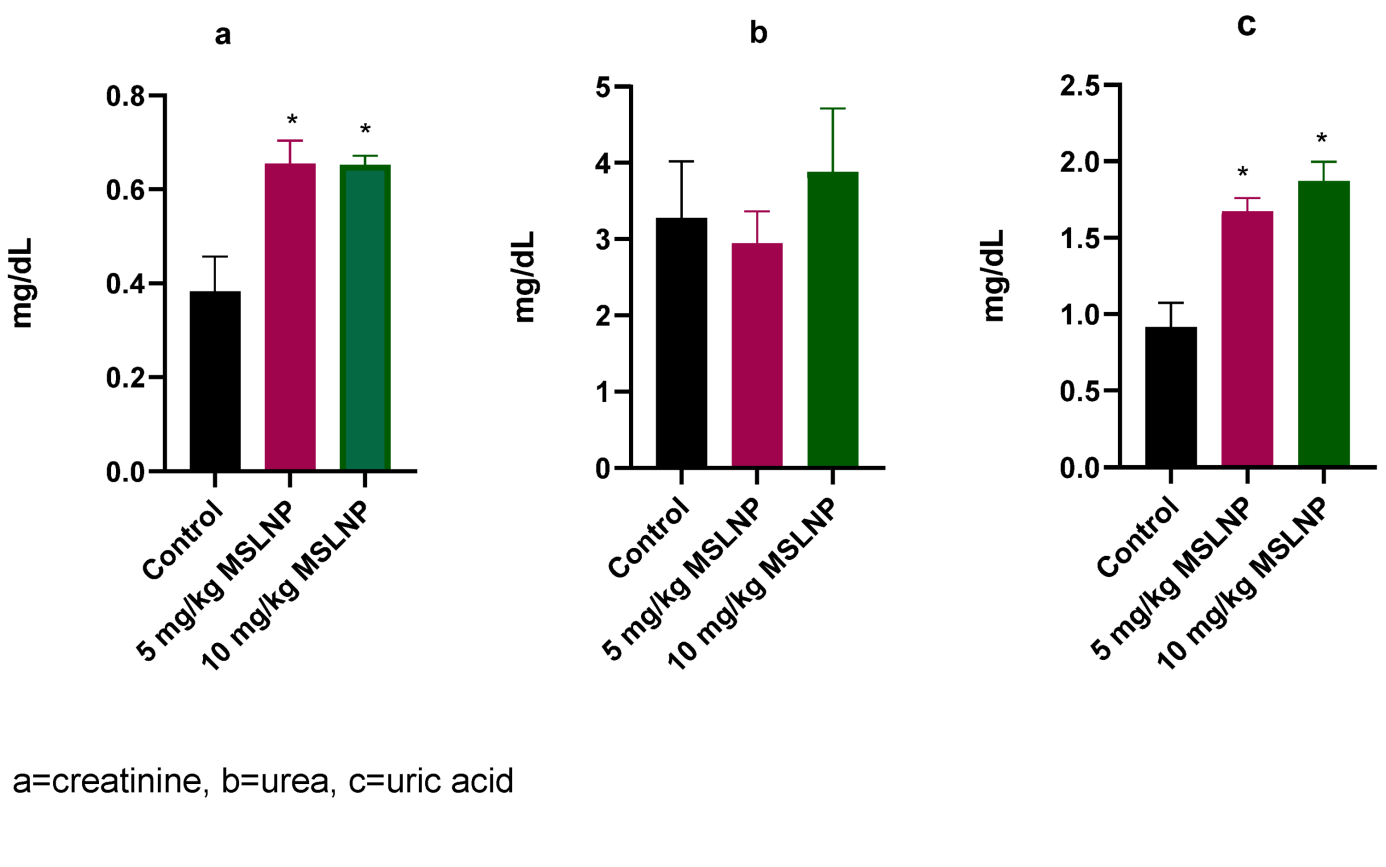
Effects of MSLNP on plasma kidney function parameters (mean and standard deviation of 7 animals; * values differ significantly from the control P<0.05)

**Figure 3:**
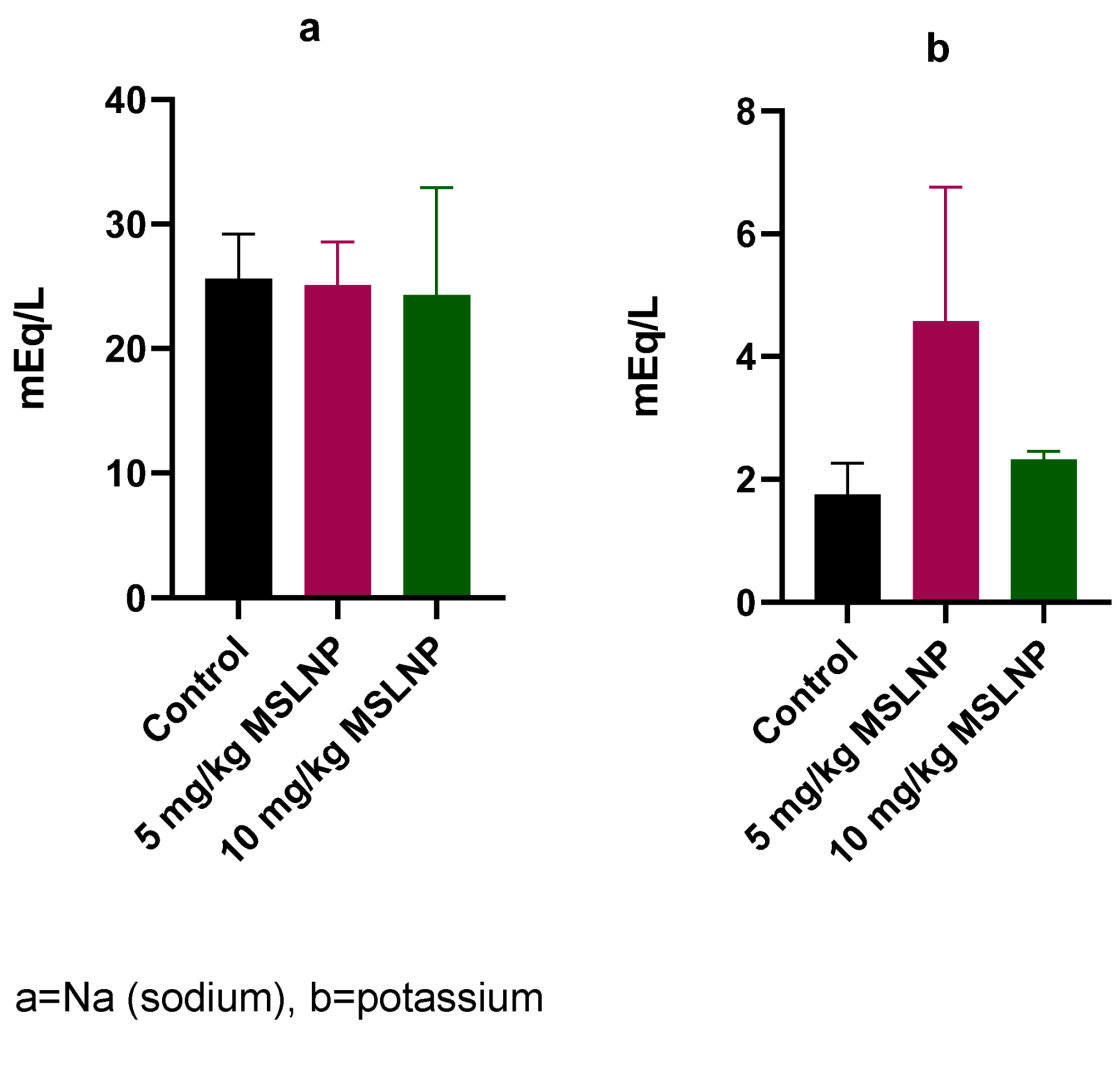
Effects of MSLNP on plasma sodium and potassium (mean and standard deviation of 7 animals

Administration of MSLNP did not produce any significant changes in plasma creatinine and urea levels in rats (Figure 2 (Fig. 2)). In contrast, there were significant elevations (p<0.05) in plasma uric acid levels in rats exposed to 5 mg/kg and 10 mg/kg of MSLNP. Furthermore, MSLNP produced non-significant changes in plasma sodium and potassium levels (Figure 3 (Fig. 3)).

**Figure 4:**
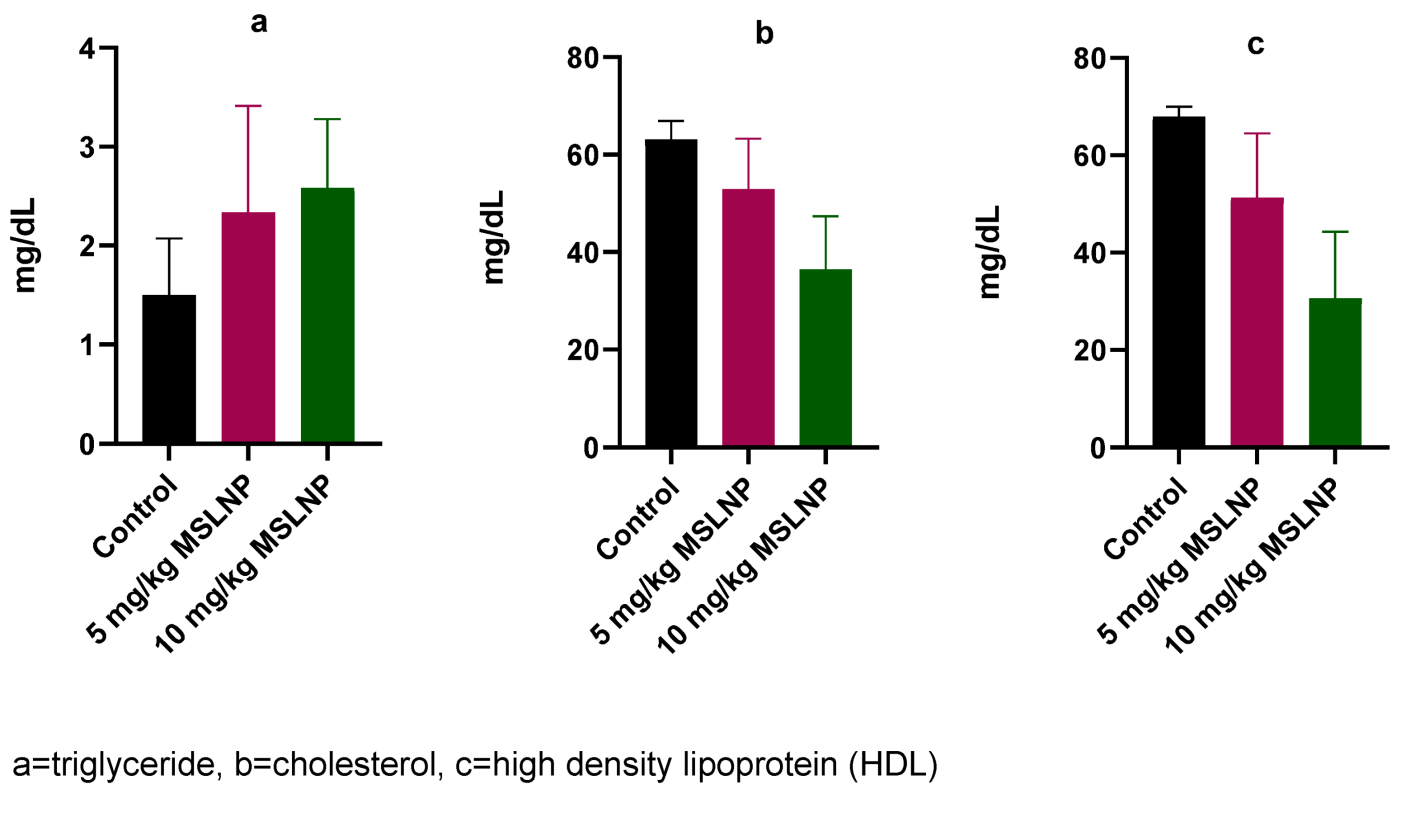
Effects of MSLNP on plasma lipid profile indices (mean and standard deviation of 7 animals)

The levels of plasma-lipid profile indices in rats exposed to MSLNP showed no significant changes in triglyceride, cholesterol and HDL compared to the control group (Figure 4 (Fig. 4)).

**Figure 5:**
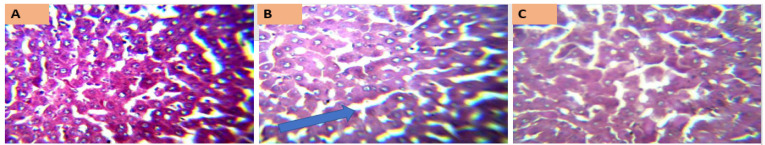
Photomicrographs of liver sections of Wistar rats exposed to MSLNP; blue arrows: sinusoids (PAS*x40**; *periodic acid-Schiff staining method; **magnification of lens)

Figure 5 (Fig. 5) depicts liver histology photomicrographs of rats exposed to MSLNP and stained with periodic acid-Schiff. Control group shows normal histoarchitecture with well-defined hepatocytes and sinusoids. The 5 mg/kg and 10 mg/kg groups did not show derangements in normal histoarchitecture, as shown by the near normal sinusoids (shown in blue) and observably distinct hepatocytes. The blue arrows in the images point to sinusoids, which are a type of blood vessel that is found in the liver. Sinusoids serve to transport nutrients and waste products between the liver and the bloodstream. The images reveal that the sinusoids in the liver are enlarged and distorted; this indicates that the liver cells are not functioning properly. The images further reveal an increase in the number of inflammatory cells in the liver, which signifies the efforts of the liver to repair itself.

**Figure 6:**
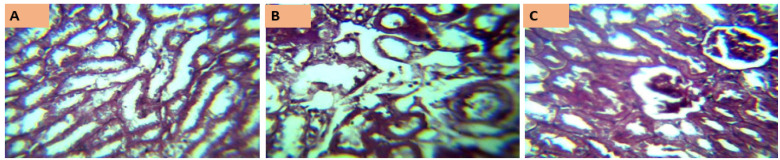
Photomicrographs of kidney sections of Wistar rats exposed to MSLNP (PAS*x40**; *periodic acid-Schiff staining method; **magnification of lens)

Figure 6 (Fig. 6) represents the kidney histology photomicrographs of rats exposed to MSLNP and stained with periodic acid-Schiff. Section A shows a relatively uniform structure, with well-defined tubules and minimal cellular damage, suggesting either a control group or low concentration exposure. Section B exhibits noticeable structural disorganization and potential cellular necrosis, indicating a moderate level of damage due to MSLNP exposure. Section C reveals pronounced tissue damage and disintegration, consistent with a high concentration of or prolonged exposure to MSLNP.

The control group shows normal kidney histoarchitecture with well-delineated glomeruli, distal and proximal convoluted tubules. There are no significant derangements observed in the histoarchitecture of other experimental groups. 

The FTIR reveals that the bands at around 3,000–2,800 cm^–1^ correspond to C–H stretching vibrations. The bands at around 1,700–1,600 cm^–1^ correspond to C=O stretching vibrations. The bands at around 1,500–1,400 cm^–1^ correspond to N–H bending vibrations. The presence of these functional groups suggests that MSLNP is a complex molecule that contains a variety of different chemical groups. This complexity may have contributed to the toxicity of MSLNP observed in the liver and kidney tissues.

## Discussion

The isolated biofilm microorganisms from the oral cavities of pupils from 5 schools across southwestern Nigeria include *P. aeruginosa*, *Aeromonas caviae*, *P. mirabilis*, and *S. mercescens*. These were the biofilm-producing isolates that passed the molecular identification criteria.* P. aeruginosa* was the predominant bacterium. A total of 10 microorganisms had a susceptibility to titanium ferrite nanoparticles coupled with *Macrosphyra longistyla* leaf extract. Out of the 10 bacteria sequenced, 7 passed the molecular identification parameter. *P. aeruginosa* is implicated in cystic fibrosis, a condition difficult to treat in children of school age and older patients. *Aeromonas caviae* is commonly isolated from water and seafood. It is also implicated in gastroenteritis in children and secretes a toxin [33]. *P. mirabilis* is a swarming biofilm-producing bacterium that could also be isolated from the oral cavity, and could be an environmental contaminant. It is also a causative agent for urinary tract infections; it is proven to be community-acquired and can lead to bacteraemia [34]. *S. marcescens* is an opportunistic biofilm-producing microorganism, which can also be antagonistic to other biofilm-producing microorganisms. This phenomenon was observed in the preliminary stages of this research (unpublished), where in several instances, two distinct bacteria were cultured on the same plate and one inhibited the growth of the other. 

Antimicrobial activity against these microorganisms showed them to be moderately to highly susceptible to the complex. The zones of inhibition show the complexes to be similar to commonly used effective antibiotics. However, the isolated bacteria possess resistant genes to these antibiotics, suggesting the antibiotic potential of the complex. The use of *Macrosphyra longistyla* and titanium ferrite against these organisms from the zone of inhibition shows it is a promising solution for inhibiting the growth of the bacteria used in this study. The results presented above show lower MICs than found in a similar study by Durugbo et al. [35]. This suggests the potential of the nanoparticle-plant extract complex as an anti-biofilm agent in the search for novel bioactive agents for use in oral health care. The MIC also aligns with those of commonly used antibiotics, to which our molecular characterisation showed the isolates as being susceptible (see Table 3 (Tab. 3)). These proved to contain several antimicrobial resistance genes, determined in-silico. These show them similar to those of similar antibiotics used in current solutions to the global problem of antimicrobial resistance. Our result, that *Macrosphyra longistyla* and titanium ferrite inhibit the growth of the bacteria tested, shows their promise as new compounds to deal with resistant strains. Hence, this study proposes a novel system of tackling AMR caused by these organisms is proposed. However, it was necessary to examine the toxic potential of this antimicrobial.

Growth parameters are commonly examined in toxicological research and help to interpret the toxicological effects of substances. The growth parameters examined here included liver and kidney weights, relative liver and relative kidney weights. Administration of *Microsphyra* *longistyla* titanium ferrite nanoparticles (MSLNP) did not produce any significant changes in the weight or relative organ weights of the rats. Akagi et al. [36] reported a similar result, in which repeated oral administration of titanium dioxide nanoparticles did not cause any significant changes on weights of liver, kidney and spleen. However, in a different study by Lin et al. [37], food-based titanium dioxide nanoparticle (anatase) was found to elicit toxic effects at dosage of 1,000 mg/kg. This demonstrates that MSLNP1 has no toxic effects on growth parameters at dosages below 10 mg/kg,

A major reason why many clinical trials of drugs fail is liver toxicity [38]. Hepatotoxicity results in development of abnormalities, which can be revealed via the determination of the levels of certain liver function serological markers [39]. The effect of administration of MSLNP on rat-liver function parameters did not elicit any significant changes in AST, ALT, ALP, bilirubin levels. However, there was a significant elevation in albumin levels in the group that received 10 mg/kg MSLNP. This result shows that MSLNP might be non-toxic to the liver at concentration below 10 mg/kg. However, Shirdare et al. [40] reported that titanium oxide nanoparticles increased the level of the hepatic enzymes AST, ALT and ALP at dosages above 300 mg/kg, which might be due to administration of titanium oxide nanoparticles at a higher dosage. 

MSLNP also showed no significant changes in kidney function parameters, which include creatinine, urea, uric acid, sodium and potassium. This agrees with the results of a study by Salehi et al. [41] in which chitosan-loaded iron-oxide nanoparticles did not elicit any significant differences in serum urea, uric acid and creatinine levels at dosages of 50, 100 and 150 mg/kg. This study shows that MSLNP might not have any toxic effects on the kidney at the given concentrations.

Photomicrographs of the liver and kidney of rats administered MSLNP did not demonstrate any deviation from normal tissue histoarchitecture. This suggests that MSLNP has no toxic effects on the liver and kidney within the administered doses. Similar results were observed by Volkovova et al. [42] with titanium dioxide and iron oxide nanoparticles. 

## Conclusion

This study has demonstrated the antimicrobial potential of nanoparticles and plant extracts against oral biofilm-producing bacteria in children. Positive reactions and low MICs show great promise of the compounds. However, additional studies are required to further concentrate the compounds in the drug design process. Further investigations are required to determine the cytotoxic effects of MSLNP, dose-response relationships, and potential mechanisms of cellular damage. 

## Notes

The co-author Professor Ernest Durugbo passed away on July 15, 2023. His contributions to the study’s design and methodology were integral to this work.

### Competing interests

The authors declare that they have no competing interests. 

### Author’s ORCID 

Nwankwo CE: https://orcid.org/0000-0003-0206-8589

### Ethical approval 

Ethical approval was obtained from Redeemer’s University Directorate of Research, Innova-tion and Partnerships, Osun Local Government, Lagos Education Board, Ibadan State Univer-sal Basic Education Board.

## Figures and Tables

**Table 1:**
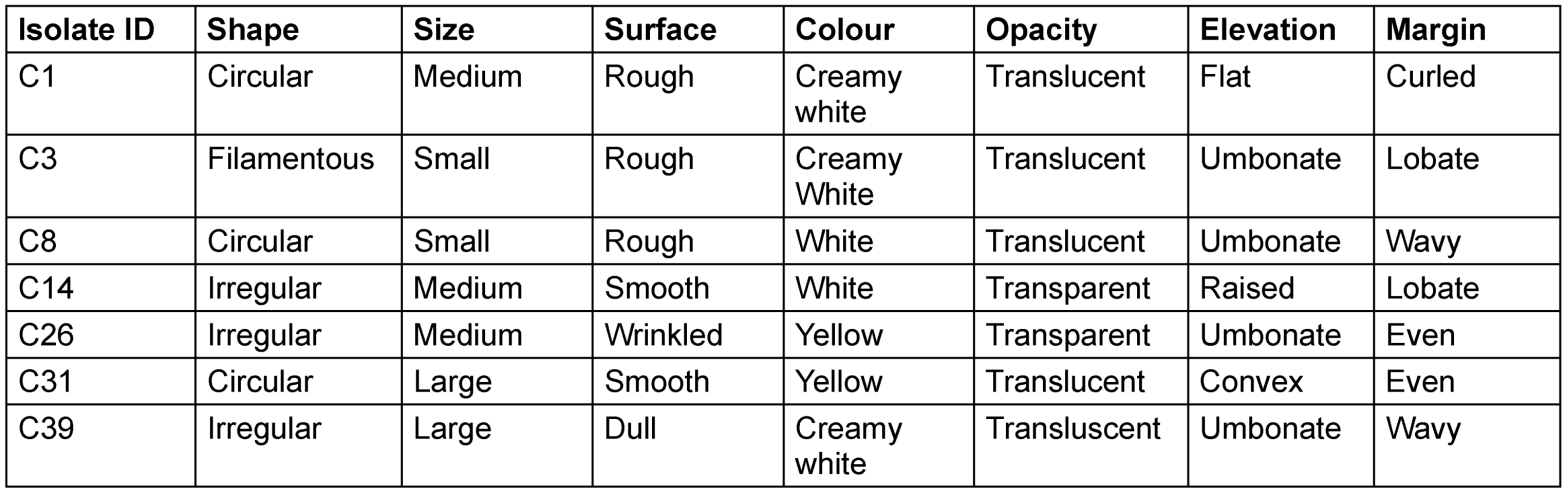
Morphology of isolates

**Table 2:**
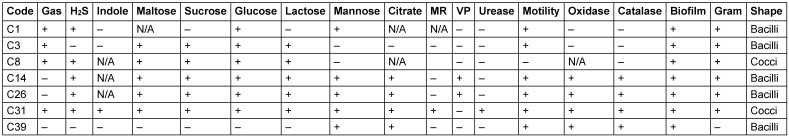
Biochemical characterization of the biofilm producing isolates

**Table 3:**
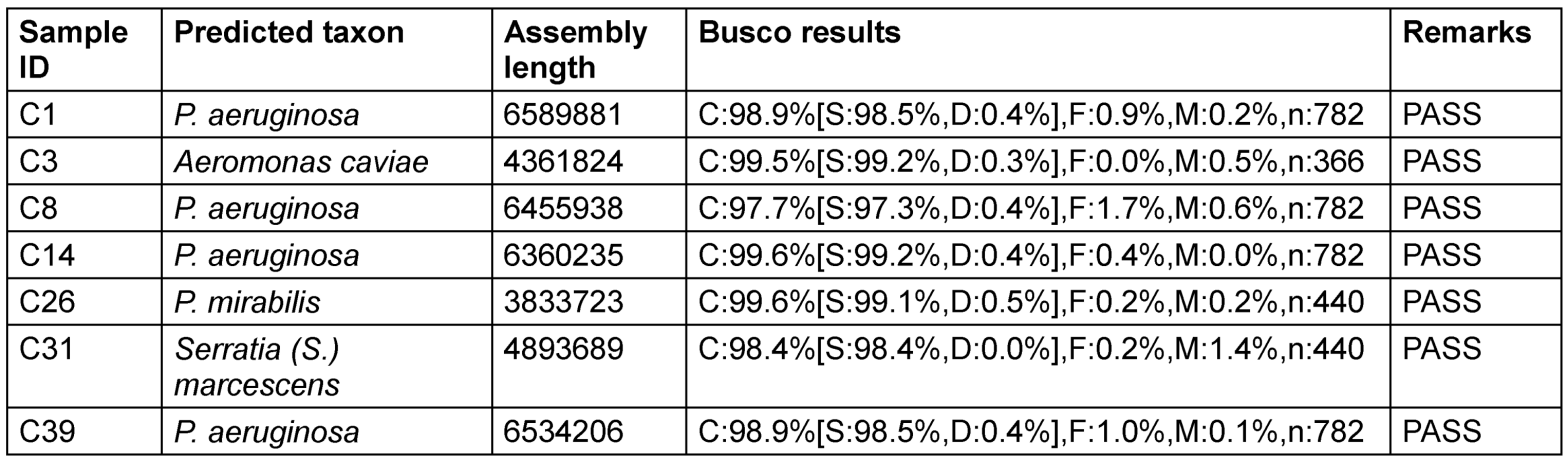
Genomic identification of isolates

**Table 4:**
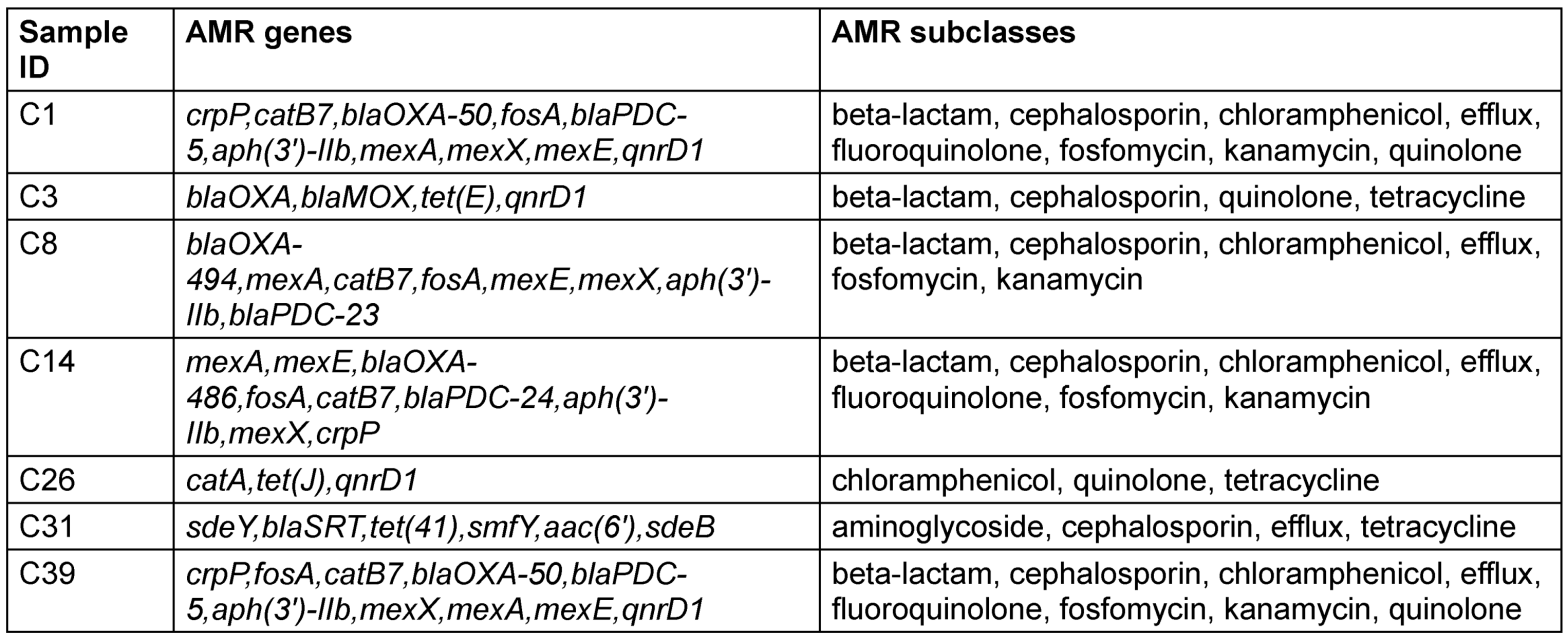
Antimicrobial resistance profile of biofilm-producing isolates

**Table 5:**
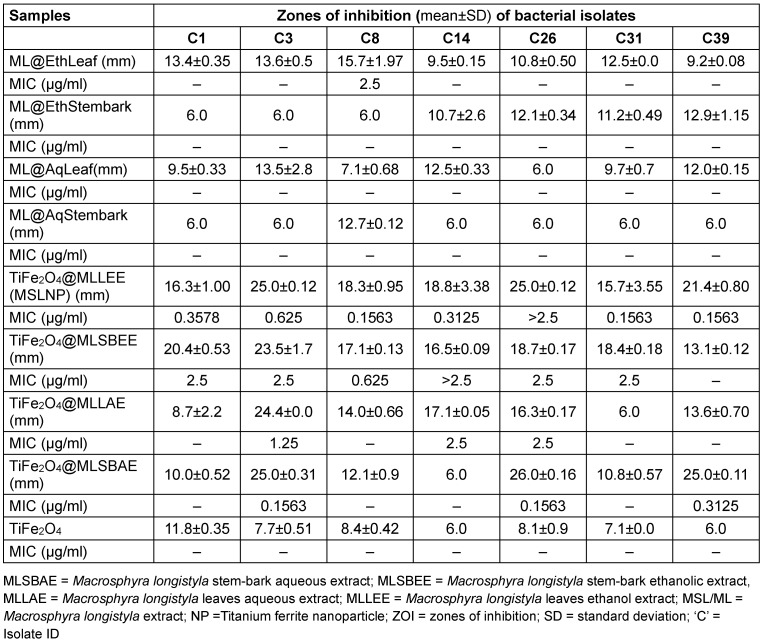
Antimicrobial susceptibility screening

**Table 6:**
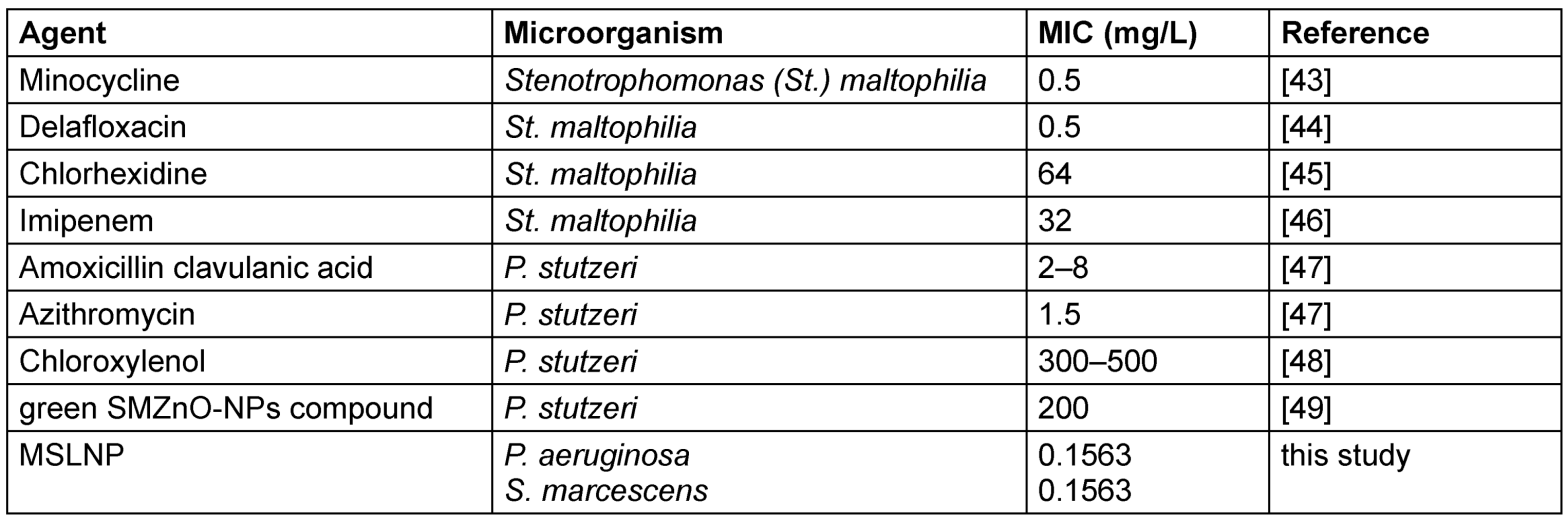
Comparison of MIC of most active compound with previous studies

**Table 7:**
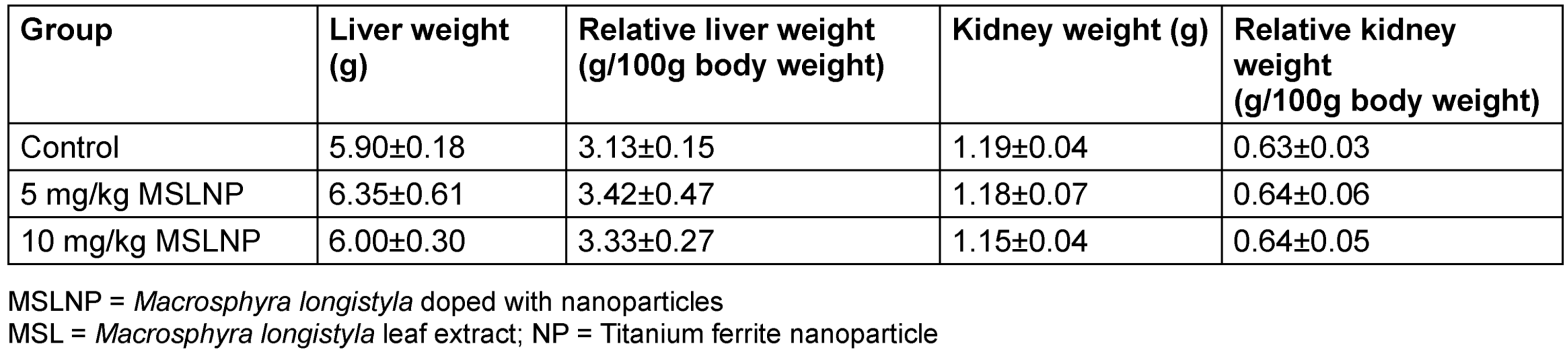
Effects of MSLNP on organ and relative organ weights of liver and kidney (mean and standard deviation of 7 animals)
